# Arterial Injury and Endothelial Repair: Rapid Recovery of Function after Mechanical Injury in Healthy Volunteers

**DOI:** 10.1155/2014/367537

**Published:** 2014-01-28

**Authors:** Lindsey Tilling, Joanne Hunt, Ann Donald, Brian Clapp, Phil Chowienczyk

**Affiliations:** Cardiovascular Division, King's College London, St Thomas Hospital, Lambeth Palace Road, London SE1 7EH, UK

## Abstract

*Objective*. Previous studies suggest a protracted course of recovery after mechanical endothelial injury; confounders may include degree of injury and concomitant endothelial dysfunction. We sought to define the time course of endothelial function recovery using flow-mediated dilation (FMD), after ischaemia-reperfusion (IR) and mechanical injury in patients and healthy volunteers. The contribution of circulating CD133^+^/CD34^+^/VEGFR2^+^ “endothelial progenitor” (EPC) or repair cells to endothelial repair was also examined. *Methods*. 28 healthy volunteers aged 18–35 years underwent transient forearm ischaemia induced by cuff inflation around the proximal biceps and radial artery mechanical injury induced by inserting a wire through a cannula. A more severe mechanical injury was induced using an arterial sheath and catheter inserted into the radial artery of 18 patients undergoing angiography. *Results*. IR and mechanical injury produced immediate impairment of FMD (from 6.5 ± 1.2% to 2.9 ± 2.2% and from 7.4 ± 2.3% to 1.5 ± 1.6% for IR and injury, resp., each *P* < 0.001) but recovered within 6 hours and 2 days, respectively. FMD took up to 4 months to recover in patients. Circulating EPC did not change significantly during the injury/recovery period in all subjects. *Conclusions*. Recovery of endothelial function after IR and mechanical injury is rapid and not associated with a change in circulating EPC.

## 1. Introduction

Arterial injury occurs in response to a wide variety of insults, including pathophysiological factors such as oxidised low-density lipoproteins, renin-angiotensin axis, and insulin resistance. In addition, iatrogenic stimuli such as instrumentation of blood vessels may lead to mechanical trauma to the endothelium. Endothelial dysfunction is thought to play a pivotal role in the pathogenesis of atherosclerosis. Vasomotor function of the endothelium, mediated mainly by nitric oxide (NO) and widely accepted as a measure of generalized endothelial cell function, is readily determined by flow mediated dilation (FMD). FMD is impaired in the presence of cardiovascular risk factors or established cardiovascular disease and is prognostic for cardiovascular events in such subjects [1–3]. Whilst much is known regarding the influence of chronic risk factors on the endothelium, relatively little is known about the response of the endothelium *in vivo* in humans to an acute injury, whether this is due to ischaemic or mechanical injury. Two previous studies have suggested a protracted course of recovery of endothelial function following instrumentation during diagnostic angiography undertaken via the radial artery. Madssen et al. found that the lumen of the cannulated artery was narrower than that of the noncannulated artery at 10–12 months after procedure [4], while Burstein et al. noted profound FMD impairment persisting at 9 weeks after procedure [5]. However, both experiments were conducted in patients with coexisting endothelial dysfunction, and the results may be attributed to a degree of trauma.

It has been suggested that endothelial health is maintained by a population of cells thought to derive from the bone marrow, termed endothelial progenitor cells (EPC). EPC appear to have a role in arterial repair and it is notable that circulating levels of EPC do predict future cardiovascular events [6], lower levels being associated with a higher event rate. However, the progenitor origin of EPC and their role in endothelial repair remain controversial.

The objectives of this study were firstly to define the time course of endothelial repair (as measured by FMD) following ischaemia-reperfusion (IR) and mechanical injury. IR and minor mechanical injury were studied in healthy volunteers and the effects of a potentially more severe mechanical injury were examined in patients undergoing invasive testing for coronary artery disease. The second objective was to examine the potential contribution of CD133^+^/CD34^+^/VEGFR2^+^ EPC to arterial healing.

## 2. Materials and Methods

All studies were approved by the local research ethics committee, and all participants provided written informed consent. Participants were asked to refrain from exercise, alcohol, and caffeine intake for 24 hours prior to the study, which took place at 9 am in a quiet temperature controlled vascular laboratory, with the subject in a supine position. Patients who had taken vasodilator medication on the morning of the study were excluded.

### 2.1. Ischaemia-Reperfusion and Mechanical Injury in Healthy Volunteers

Subjects were healthy volunteers aged 18–35 years with no known risk factors for coronary artery disease and were on no regular medications. Anthropometric characteristics were recorded and seated blood pressure and pulse rate were taken as the mean of three sequential readings using an oscillometric monitor (Omron) following 15 minutes of rest. Blood was collected via peripheral venous sampling at the antecubital fosse for biochemical characterization and measurement of baseline circulating EPC. FMD (ipsilateral to the site of interventions on the arm) and circulating EPC (contralateral to the site of interventions on the arm) were measured before and at serial time points after each of the following interventions: IR of the forearm and mechanical injury of the radial artery.

### 2.2. IR of the Forearm in Healthy Volunteers (*n* = 10)

Transient forearm ischaemia was induced by inflating a cuff on the right upper arm around the proximal biceps to at least 50 mmHg above systolic pressure for 20 minutes. This period of ischaemia, when followed by reperfusion, is known to elicit transient endothelial dysfunction [7]. Blood samples were taken at baseline from the left antecubital fossa and again 10 minutes, 1 hour, 2 days, and 7 days after the procedure. FMD was measured in the right arm at baseline and again 10 minutes, 6 hours, 2 days, and 7 days after IR.

### 2.3. Mechanical Injury of the Radial Artery in Healthy Volunteers (*n* = 18)

Following the demonstration of a normal Allen's test, venous blood was sampled (left arm) and FMD (right arm) was measured as in the IR protocol. A 20-gauge arterial cannula (Vygon) was then inserted into the volar aspect of the right radial artery, at a level within three finger-breadths of the radial styloid process, using a sterile technique and 2 mls of 1% lignocaine for local subcutaneous anaesthesia. The cannula was inserted using a Seldinger technique: a 20-gauge introducer needle (3 cm length, 0.90 mm diameter) was inserted into the radial artery at an angle of 45° through which a 20 cm guidewire of 0.53 mm diameter was introduced. Only a single pass of the wire through the needle was made. The introducer needle was removed and a plastic cannula sheath was “fed” over the guide wire. To encourage deendothelialisation, a 30° kink was then formed in the guide wire at 2 cm from the tip. It was reinserted through the cannula, and rotated through 360°. The guide wire was then removed and the cannula was flushed with 5 mls of normal saline. The cannula was left in situ for five minutes and then removed. Blood was sampled at baseline and again 10 minutes, 1 hour, 2 days and 7 days after the injury. FMD of the injured arm was performed at baseline, 10 minutes, 6 hours, 2 days, and 7 days.

### 2.4. Mechanical Injury of the Radial Artery in Patients (*n* = 18)

In order to examine the effect of a larger vascular injury on FMD and EPC level, patients undergoing investigation for possible coronary artery disease were examined before and after radial artery injury caused by instrumentation of the radial artery for diagnostic angiography. Patients had been referred to the General Cardiology Clinic at Guy's Hospital regarding symptoms suggestive of coronary disease. All patients had consented to having the angiogram performed from the radial artery in the wrist and had a normal Allen's test. The sheath employed in gaining access for radial angiography is thought to cause limited, local trauma to the artery and hence provide a greater degree of injury than that provoked by the wire used in healthy volunteers. Patients were aged between 40 and 75 years. Exclusion criteria included acute coronary syndrome or percutaneous/surgical intervention in the past three months, simultaneous angioplasty, previous radial artery cannulation, previous coronary artery bypass grafting involving use of the radial artery, New York Heart Association (NYHA) class III/IV heart failure, serum creatinine >140 *μ*mol/L, malignancy in the past ten years, active inflammation, severe chronic obstructive pulmonary disease, and hepatic failure. Blood pressure was recorded for the healthy volunteers, and venous blood was taken from the antecubital fossa.

The radial sheath was inserted using a Seldinger technique into the volar aspect of the right radial artery, at a level within 3 finger-breadths of the radial styloid process, using a sterile technique and 2 mls of 1% lignocaine for local subcutaneous anaesthesia. A 21-gauge introducer needle (4 cm length, 0.82 mm diameter, Cook Medical) was first inserted into the radial artery at an angle of 45° through which a 40 cm guidewire of 0.46 mm diameter was introduced. A 5 French sheath (13 cm length, l.67 mm diameter, Cook Medical) was fed over the wire, which was then removed. A bolus of glycerol nitroglycerin (GTN, 1 mg diluted to 5 mL with normal saline) was then injected intra-arterially through the side port of the sheath to try and prevent arterial spasm. The catheter was then introduced, and 2000-unit heparin administered intra-arterially at the level of the aortic arch. The angiogram was then carried out as per usual. At the end of the procedure, the sheath was removed and haemostasis was achieved with the use of a compression assist device. FMD was performed on the right arm along with blood sampling of the uninjured arm before the angiogram (baseline) and again at 2 days (*n* = 18), 7 days (*n* = 11), 1 month (*n* = 11), and 4 months (*n* = 10).

### 2.5. Laboratory Studies

Venous blood was collected for laboratory tests including full blood count, lipid profiles, and renal function. EPC analysis was performed using flow cytometry as previously described [8]. Whole blood collected in EDTA-containing tubes was spun down and the plasma discarded. Fc receptor blocker (MACS Miltenyi Biotech, Surrey, UK) was added to each sample, before incubation on ice. Antibodies to Peridinin chlorophyll protein (PerCP)conjugated CD34 (BD Biosciences, Oxford, UK), Phycoerythrin (PE)conjugated CD133 (MACS Miltenyi Biotec), and Fluorescein isothiocyanate (FITC)conjugated VEGFR2 (R&D, Abingdon, UK) were added to identify EPC. Following red cell lysis with lysis buffer (Ebioscience, Hatfield, UK) the samples underwent further centrifugation before resuspension of the pellet in phosphate buffered saline with 0.5% newborn calf serum. Cells were acquired (1 × 10^5^) using a Becton Dickinson FACSCanto II flow cytometer and analysed using Diva software (BD Biosciences) and they were expressed as a fraction of the mononuclear cell population. All cell population measurements were carried out in duplicate to assess reproducibility.

### 2.6. Endothelial Function

Endothelial NO-dependent vasomotor function was assessed by measuring FMD of the radial artery according to current guidelines [9]. High resolution ultrasound (Siemens Aspen with 7 MHz linear array transducer, positioned by a stereotactic manipulator) was used to scan the radial artery in a longitudinal section. After optimal positioning of the transducer, a baseline scan was recorded. An increase in flow was then induced by inflation of a pneumatic tourniquet placed around the arm (distal to the arterial segment being scanned) to a pressure of 250 mmHg for 5 minutes, followed by release. A second scan commenced 10 seconds before the release of the cuff and was continued for 3 minutes after cuff deflation. After 10 minutes of vessel recovery, another resting scan was taken. Sublingual GTN (25 *μ*g) was then administered (as an endothelium-independent control), and a final scan was performed 3 to 4 minutes later. Images were digitized for subsequent blinded analysis using automated edge detection software (Brachial Analyser, Medical Imaging Applications, LCC, IA, USA). FMD was expressed as the percentage increase in radial artery diameter from baseline to maximal dilation which occurred 30 to 90 seconds after the release of the cuff. Dilation to GTN was expressed as the percentage increase in radial artery diameter from baseline to maximal dilation after GTN. The standard deviation of within-subject between-visit differences in FMD in our laboratory is less than 2%.

### 2.7. Statistical Analysis

Subject characteristics are expressed as mean ± SD and results as mean ± SE (or as median and interquartile range, IQR). Outcome measures (change from baseline) were compared using ANOVA for repeated measures. Prespecified contrasts were used to test for differences relative to baseline at specific time points. In the study on patients, not all participants attended all study time points. We therefore imputed the mean for three EPC and four FMD values to provide the missing data. All tests were 2-tailed and *P* < 0.05 was taken as significant. SPSS version 16 was used for all tests.

## 3. Results

28 healthy volunteers (4 female) aged 25.7 ± 7.1 years and 18 patients (7 female) aged 60.6 ± 8.8 years were enrolled. Baseline characteristics are shown in Table 1. The patient cohort had an average of BMI > 30, and one-third were diagnosed with diabetes. However, their blood pressure and total cholesterol level were well controlled. 6/18 (33.3%) were newly diagnosed with coronary artery disease at angiography.

### 3.1. Ischaemia-Reperfusion and Mechanical Injury in Healthy Subjects

**Figure 1:**
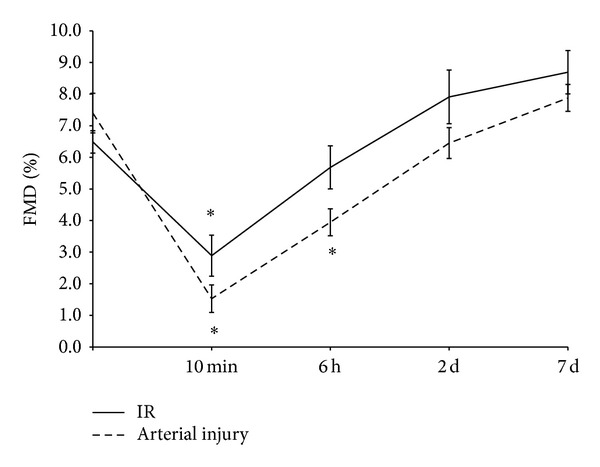
Flow mediated dilation (FMD) of the radial artery following arm ischaemia-reperfusion (IR) and radial injury, *P < 0.001.

Forearm IR and mechanical injury to the radial artery were both associated with a transient impairment of FMD of the radial artery which was more marked and of longer duration after mechanical injury than IR (Figure 1). FMD decreased from 6.5 ± 1.2% at baseline to 2.9 ± 2.2% at 10 minutes after IR and from 7.4 ± 2.3% at baseline to 1.5 ± 1.6% at 10 minutes after arterial injury (both *P* < 0.001). At 6 hours, FMD after IR was not significantly different from baseline (5.7 ± 2.3%) but remained depressed after mechanical injury (3.9 ± 1.5%, *P* < 0.001). At 2 days after both interventions FMD was similar to baseline.

### 3.2. Mechanical Injury in Patients

**Figure 2:**
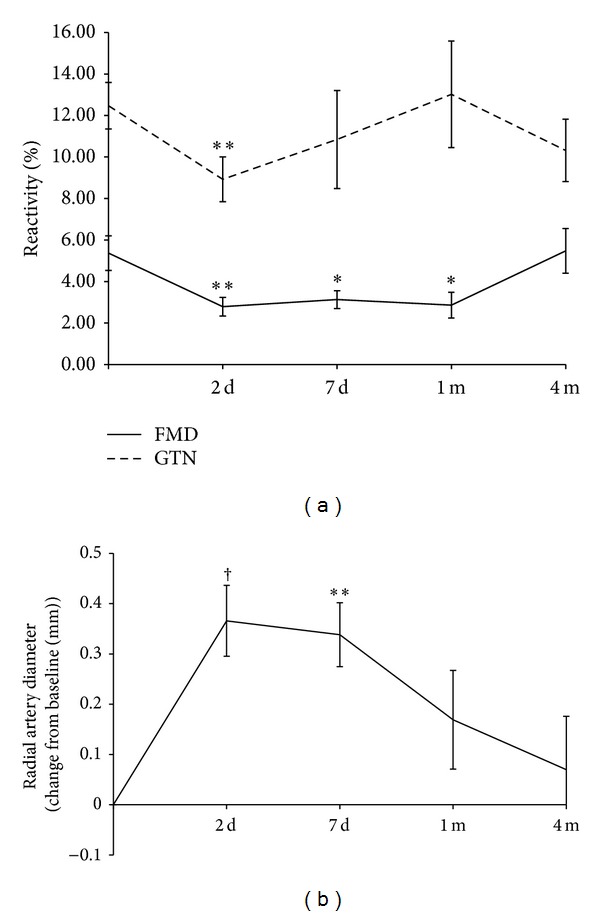
(a) Flow mediated dilation (FMD) and response to nitroglycerine (GTN) of the radial artery following radial sheath insertion; (b) change from baseline in radial artery diameter following radial sheath insertion; *P < 0.05, **P < 0.01, and ^†^ *P* < 0.001.

Following radial artery sheath insertion in patients undergoing angiography, there was an early, significant impairment of FMD (5.4 ± 4.0% at baseline versus 2.8 ± 2.1% at day 2, *P* = 0.0021), which was sustained at 1 month but had recovered by 4 months (Figure 2(a)). The response to GTN was impaired 2 days after angiography (12.5 ± 5.4% at baseline versus 8.9 ± 5.1% at day 2, *P* = 0.0013) but returned to baseline 7 days after angiography (Figure 2(a)).

Radial artery diameter increased from baseline after angiography from 2.58 ± 0.4 mm at baseline to 2.91 ± 0.5 mm at day 2 and 2.80 ± 0.3 mm at day 7 (both *P* < 0.01, Figure 2(b)) but was not significantly different from baseline at 1 and 4 months after angiography.

### 3.3. Endothelial Progenitor Cells

There was no consistent effect of IR or mechanical injury on EPC in healthy volunteers. Mechanical injury of the radial artery in patients also produced no significant effect on EPC (Table 2).

## 4. Discussion

These experiments examined the relationship between the separate insults of IR and mechanical injury on vasomotor function and CD133^+^/CD34^+^/VEGFR2^+^ EPC in healthy subjects and patients under investigation for coronary artery disease. The time course of arterial repair following local mechanical injury is unknown. Previous studies in patients undergoing radial sheath insertion suggest a protracted recovery period but may be confounded by the presence of cardiovascular disease, drugs, and mechanical injury to the underlying smooth muscle. The radial artery is increasingly harvested and used in coronary artery bypass surgery. As diagnostic angiography and percutaneous coronary intervention are frequently performed from the wrist, this has implications for later use of the radial artery as a bypass conduit. A study which examined structural and functional changes to the radial artery at 10–12 months after angiography found the lumen of the cannulated artery was narrowed in comparison to the non-cannulated artery. No difference was found in vasomotor function assessed by FMD between the cannulated and non-cannulated arteries [4]. Another study looked at the changes 24 hours and 9 weeks after radial cannulation for angiography. A profound impairment of FMD was observed at 24 hours, which persisted at 9 weeks, in accordance with our results [5]. A reduction in the response to nitrate was also noted, in agreement with our data which showed a significant reduction in GTN response at 2 days after sheath insertion. This suggests that not only is endothelial function affected by local trauma, but also that there is smooth muscle cell dysfunction that may contribute to the immediate impairment of FMD.

Burstein et al. also documented a significantly larger radial artery diameter after procedure. This increase in diameter was also seen in this group of patients at 2 and 7 days and may reflect mechanical trauma by the sheath to the vessel wall, consistent with the attenuated GTN response. Any change in diameter may influence the subsequent calculation of FMD. However, that the changes in FMD are not simply a function of augmented radial artery diameter is apparent from the difference in chronology. FMD remains significantly depressed at 1 month, whereas the radial artery diameter returns to baseline after 1 week.

This is the first study to examine recovery of endothelial function after mechanical injury in healthy subjects and the first demonstration *in vivo* in humans of rapid recovery. We have shown that both IR and injury result in a significant but transient vascular dysfunction manifest by an immediate reduction in FMD, the size and duration of which proportional to the degree of insult, such that ischaemia-reperfusion was less than wire-mediated injury which itself was less than sheath-mediated injury.

Endothelial ischaemia and injury were not associated with a consistent change in EPC. This suggests that these cells are not wholly responsible for the relatively rapid improvement in endothelial function seen in these experiments. Few studies have examined the effect of isolated IR or injury on EPC. Friedrich et al. observed forearm IR in healthy subjects and found an association with reduced CD34^+^/CD133^+^ EPC immediately and 2 hours after IR [10]. They speculated this may represent increased EPC adhesion to the ischaemic vasculature or differentiation of this cell population into a more mature cell type. However, murine studies of acute limb IR have shown an elevation in EPC numbers [11], and repetitive ischaemic preconditioning in humans increased CD34^+^/CD133^+^/CD45^low^ EPC [12]. Many studies have demonstrated an increase in EPC of varying phenotypes, following the combined ischaemia/injury insult seen after coronary stenting [13], in experimentally induced myocardial infarction [14] and in pathological myocardial infarction [15]. These are admittedly much larger stimuli, which may also be complicated by the presence of inflammation. It is important to note that cellular and molecular mechanisms of neovascularisation differ depending on whether a denuded artery, hindlimb ischaemia, MI, or a traumatic injury is studied [16].

It is probable that other circulating cell subtypes are involved in endothelial repair, and there is a large amount of ongoing research in this area. Like EPC, circulating endothelial cells express CD34 and VEGFR2 but do not express CD133 or CD45. Circulating endothelial cells have been said to differ fundamentally from progenitors in that they have no proliferative capacity; however, they may play a part in structural repair of the damaged endothelium. Endothelial colony forming cells are a rare circulating endothelial cell subtype which have robust clonal proliferative potential and can form secondary and tertiary colonies upon replating. They have been shown to form intrinsic *in vivo* vessels upon transplantation into immunodeficient mice and are another cell thought to have a role in endothelial repair [17].

As well as indicating the presence of dysregulation, various biomarkers are now thought to play an active part in the repair of the endothelium. One such biomarker which has been under recent scrutiny is microRNA. MicroRNAs are short, highly conserved noncoding sections of RNA, 20–25 nucleotides long, which regulate protein synthesis at the posttranscriptional level. They were initially used as biomarkers in cancer diagnosis and prognosis; however, certain circulating microRNAs have now been explored as biomarkers for coronary artery disease. Some microRNAs are specific to the endothelium and may be proangiogenic. An example is microRNA-210, known to be induced by endothelial hypoxia and recently shown to promote tube formation and endothelial cell migration [18].

A number of important limitations to this study should be noted. We studied the effects of acute interventions to the forearm and the results may not be relevant to other vascular beds. This was a single insult, and effects of chronic or repetitive ischaemia or injury may differ from these acute effects. Patients were not standardized in terms of the drugs they were taking before their procedure, and we did not control for the presence of cardiovascular risk factors (Table 1). However, each patient acted as their own control, as all measurements were compared to the individual's baseline values; therefore, this should not have affected the results. Intra-arterial GTN was administered at the start of the investigation, and contrast was used during the angiogram, but each participant underwent exactly the same procedure, and there is no data to suggest either substance would affect FMD or EPC measured at 2 days and beyond. We did not measure the flow or shear stimulus to FMD and cannot exclude the possibility that changes in FMD could be secondary to differences in the flow response. However, previous studies have shown no difference in hyperaemic shear rate after radial artery injury [19]. Finally, we studied EPC characterized by CD133^+^/CD34^+^/VEGFR2^+^ surface markers. We chose these antigens as they have been accepted as defining an EPC for many years, although they were recently suggested to be an enriched population of haematopoietic precursors, incapable of endothelial cell formation [20]. Despite this, CD133^+^/CD34^+^/VEGFR2^+^ cells have been repeatedly demonstrated to correlate with cardiovascular risk factors and outcomes, and it is still possible that these cells are mobilized to areas of tissue injury and function in a paracrine manner to facilitate repair by other local cells.

In conclusion, this is the first study to examine the time course of recovery of endothelial function following local injury to the endothelium in healthy subjects. It suggests that functional recovery is rapid, being complete within 7 days, and that EPC are unlikely to be involved in this rapid recovery.

## Figures and Tables

**Table 1:** Baseline characteristics.

	Healthy volunteers (*n* = 28)	Patients (*n* = 18)	*P* value
Age (years)	25.7 ± 7.1	60.6 ± 8.8	<0.001
Gender (male)	24 (85.7)	11 (61.1)	<0.001
Ethnicity (white)	19 (67.8)	13 (72.2)	Ns
BMI	23.9 (2.7)	31.2 (6.6)	<0.001
Systolic BP (mmHg)	131 (9.1)	127.3 (17.2)	Ns
Diastolic BP (mmHg)	75.1 (8.3)	74.4 (11.5)	Ns
Hypertension	0 (0)	10 (55.5)	<0.001
Diabetes	0 (0)	6 (33.3)	<0.001
Current smoker	0 (0)	3 (16.7)	<0.001
Concurrent medications:			
Antiplatelet	0 (0)	12 (66.7)	<0.001
Statin	0 (0)	13 (72.2)	<0.001
Cholesterol (mmol/L)	4.1 ± 1.0	4.2 ± 1.2	Ns
Glucose (mmol/L)	5.1 ± 0.8	6.7 ± 2.5	Ns
Baseline radial artery diameter (mm)	2.42 ± 0.3	2.58 ± 0.4	Ns
Baseline FMD (%)	7.0 ± 1.9	5.4 ± 4.0	Ns

Values are mean ± SD or number (%). BMI: body mass index; BP: blood pressure; FMD: flow mediated dilation; Ns: not significant.

**Table 2:** Effect of ischaemia and injury on endothelial progenitor cell (EPC) numbers.

	Healthy volunteers: IR	Healthy volunteers: injury	Patients
V/133 baseline	404 ± 38	267 ± 22	50 ± 14
2 d	465 ± 138	241 ± 27	23 ± 4
7 d	450 ± 179	270 ± 22	29 ± 13
4 m			40 ± 29
V/34 baseline	18 ± 6	11 ± 3	7 ± 2
2 d	11 ± 4	7 ± 1	7 ± 2
7 d	9 ± 2	12 ± 2	3 ± 1
4 m			2 ± 1
133/34 baseline	74 ± 14	78 ± 10	16 ± 2
2 d	73 ± 14	96 ± 13	19 ± 4
7 d	82 ± 13	105 ± 16	20 ± 4
4 m			21 ± 5

Values are mean ± SEM. IR: ischaemia-reperfusion; V/133 VEGFR2^+^/CD133^+^ cells; V/34 VEGFR2^+^/CD34^+^ cells; 133/34 CD133^+^/CD34^+^ cells.
